# Identification of Two Novel Linear Neutralizing Epitopes within the Hexon Protein of Canine Adenovirus Using Monoclonal Antibodies

**DOI:** 10.3390/vaccines9020135

**Published:** 2021-02-08

**Authors:** Shujie Wang, Chunsheng Wang, Xiao Ren, Wenjiao Xue, Haijuan He, Yanzhu Zhu, Hongfeng Wang, Gang Wang, Xuehui Cai

**Affiliations:** 1 State Key Laboratory of Veterinary Biotechnology, Harbin Veterinary Research Institute, Chinese Academy of Agricultural Sciences, Harbin 150001, China; rxbioclub@sina.com (X.R.); xue5323@yeah.net (W.X.); he_haijuan@haas.cn (H.H.); wanghongfeng01@caas.cn (H.W.); wanggang@caas.cn (G.W.); 2 College of Life Science, Northeast Forestry University, Harbin 150040, China; wangchunsheng79@nefu.edu.cn; 3 Animal Husbandry Research Institute, Heilongjiang Academy of Agriculture Sciences, Harbin 150086, China; 4 Key Laboratory of Special Animal Epidemic Disease, Ministry of Agriculture, Institute of Special Animal and Plant Sciences, Chinese Academy of Agricultural Sciences, Changchun 130112, China; zhuyanzhu@caas.cn

**Keywords:** canine adenovirus, identification, hexon protein, monoclonal antibodies, novel epitopes

## Abstract

Canine adenovirus (CAdV) has a high prevalence in canine populations. High affinity neutralizing antibodies against conserved epitopes can provide protective immunity against CAdV and protect against future outbreaks. In this study, we identified two CAdV-2-specific neutralizing monoclonal antibodies (mAbs), 2C1 and 7D7, which recognized two linear-dependent epitopes. MAb 2C1 potently neutralized CAdV-2 with a 50% neutralization titer (NT50) of 4096, and mAb 7D7 partially neutralized CAdV-2 with a 50% NT50 of 64. Immunoprecipitation, Western blot and protein spectral analysis indicated that both neutralizing mAbs recognized the hexon protein (Hex) of CAdV-2. Through a 12-mer random peptide phage display and synthetic peptides analysis, we finely mapped the neutralizing epitopes to two 10-amino acid (aa) peptides within the CAdV Hex: ^634^RIKQRETPAL^643^ located on the surface region; and ^736^PESYKDRMYS^745^ located in the inner region of the expected 3D structure of trimeric Hex. Importantly, the two epitopes are highly conserved among all CAdV isolates by sequence alignment analysis. Thus, these results provide insights into the interaction between virus and mAbs at the aa level and may have potential applications in the development of novel therapeutic or epitope-based vaccines, antibody therapeutics and a diagnostic method suitable for the rapid detection of all CAdVs.

## 1. Introduction

Infectious canine hepatitis (ICH) is an acute septic infectious disease, and its etiological agent is canine adenovirus type 1 (CAdV-1). Hepatitis, corneal oedema (blue eye), jaundice and anemia are the main clinical symptoms. Some studies showed that CAdV-1 continues to circulate in carnivores [1,2,3]. CAdV type 2 (CAdV-2) can cause infectious tracheobronchitis (ITB) and pneumonia in dogs, also known as kennel cough, fever, serous to mucinous rhinorrhea, tonsillitis and laryngotracheitis in dogs, which has a high prevalence in canine populations [4,5].

CadVs have non-enveloped capsids with pseudo-icosahedral symmetry, linear double-stranded DNA of about 30 kb and are included in the genus *Mastadenovirus* of the family *Adenoviridae* [6]. The genome of CAdV includes 30 open reading frames (ORFs) flanked by two identical 161 bp inverted terminal repeats [7]. The major coat protein of CAdV is hexon (Hex), which plays a major role in viral tropism and neutralization [8]. The N-terminal domain (with a core size ~484 amino acids [aa]) and C-terminal domain (core size ~221 aa) adopt the same PNGase F-like fold, although they are significantly different in length. The CAdV capsid structure is relatively well conserved, and is primarily composed of homo-trimers of Hex; 240 trimers form the icosahedron’s 20 facets [9,10]. There are 12 vertex penton capsomers, each with a fiber protruding from the surface [10,11,12]. The trimeric Hex has a pseudohexagonal base with three towers extending upwards [10,11]. Apart from these, the capsid is also stabilized by other Hex-associated proteins [13]. It is estimated that most of the adenoviral neutralizing antibodies are generated against Hex [14]. 

In this study, we characterized the potent neutralizing monoclonal antibody (mAb) 2C1, and a partially neutralizing mAb 7D7, raised against CAdV-2. We expressed and purified a truncated His-fused Hex (552–850 aa) containing most of C-terminal domain; then tested the neutralizing mAbs for recognition in Western blot (WB) assays. Using a phage display approach, we finely mapped the recognized epitopes to ^634^RIKQRETPAL^643^ and ^736^PESYKDRMYS^745^ on the CAdV-2 Hex. Since the two linear epitopes are highly conserved among CAdV-1 and CAdV-2 strains, these research results may assist in the design of structure-based novel epitopes vaccines or therapeutic vaccines and antibody therapeutics, and the development of rapid detection techniques for CAdV antigens or antibodies. 

## 2. Materials and Methods

### 2.1. Virus Strains, Hybridoma Lines, Plasmids and Phage Peptide Library

The CAdV-2 strain used in this study was stored at the Harbin Veterinary Research Institute of the Chinese Academy of Agricultural Science. Hybridoma lines secreting CAdV-2-specific mAbs were generated in our lab previously [15]. The pET-30a (+) vector was purchased from Tiangen, China. A commercially available Ph.D.-12TM Phage Display Peptide Library Kit was purchased from New England BioLabs Inc. CAdV-2 was cultured in Madin–Darby canine kidney (MDCK) cells in Dulbecco’s modified eagle’s medium with 10% fetal calf serum (Gibco, USA) at 37 °C, and *Escherichia coli* ER2738 and Rosetta (DE3) were cultured in Luria–Bertani (LB) medium.

### 2.2. Characterization of mAbs against CAdV-2

Hybridoma lines secreting CAdV-2-specific mAbs were cultured, and the isotype was determined using the SBA Clonotyping^TM^ System/HRP Kit (Southern Biotech, USA). Specificity characterization of CAdV-2-specific mAbs 2C1 and 7D7 was carried out by indirect immunofluorescence assay (IFA) and WB according to standard procedures [15], but WB using denaturing SDS-PAGE and native PAGE, respectively. Denaturing SDS-PAGE was carried out according to standard procedures. In brief, 15 μL of virus sample was mixed with 5 μL 4X SDS-PAGE loading buffer and boiled for 10 min. Samples were then loaded into 12% SDS-PAGE gel (Invitrogen), and electrophoresis was performed at room temperature using a constant voltage (120 V). In NSDS-PAGE, 15 μL of virus sample was mixed with 5 μL of 4X NSDS sample buffer (150 mM Tris base, 100 mM Tris HCl, 10% (v/v) glycerol, 0.0185% (w/v) Coomassie G-250, 0.00625% (*w/v*) phenol red, pH 8.5, Invitrogen). Then, samples were loaded into the gel and electrophoresis was conducted using the chilled NSDS-PAGE running buffer (50 mM Tris Base, 50 mM MOPS, 0.0375% SDS, pH 7.3) under 4 °C at 120 V [16].

### 2.3. Micro-Neutralization Assay

The micro-neutralization assay was performed according to the Office International Epizooties (OIE) manual. Briefly, ascetic fluid of 2C1or 7D7 was heat-inactivated for 30 min at 56 °C. Two-fold serial dilutions of heat-inactivated ascetic fluid of 2C1or 7D7 were mixed with an equal volume of viral solution containing 200 50% tissue culture infectious dose (TCID_50_) CAdV-2, anti-CAdV-2 mouse serum as positive control and a mAb without activity as a negative control, and then mixtures were incubated for 1 h at 37 °C in 96-well microplates; afterward, 150 μL medium containing 10^4^ MDCK cells was added to every well. The plates were incubated for 4 days at 37 °C in a humidified atmosphere with 5% CO_2_, and we inspected cytopathic effects via an inverted optical microscope. The experiment was repeated 3 times. The Kärber method was used to calculate the 50% neutralizing titer (NT50) [17,18].

### 2.4. Immunoprecipitation and MALDI-TOF-MS

In order to identify the CAdV-2 protein that could bind to the mAbs, 1 μL Triton X-100 was added in 100 μL CAdV-2 suspension from infected cells for 1 h at 4 °C; then cell lysates from infected cells were added to pre-cleared protein A/G agarose beads for 2 h, followed by incubation with purified mAb ascite fluids of 2C1 or 7D7 overnight at 4 °C. 30 μL immunoprecipitates were suspended in sample buffer and boiled for 10 min, then centrifuged and analyzed by SDS-PAGE. Protein bands were subjected to MALDI-TOF MS analysis, and mass spectra were acquired by an Ettan MALDI-TOF Pro mass spectrometer (GE Healthcare, Uppsala, Sweden) by Sensichip Infortech Co. Ltd. (Shanghai, China).

### 2.5. Expression and Identification of Truncated Hex in E. coli

According to the sequence of CAdV-2 Hex (GenBank accession number AC000020.1), the primers were designed to amplify an 897-bp fragment (F: 5′-1654-TATGAATTCGACCTCCGAGTGGATGGG-1671-3′, R: 5′-2533-GAGCTCGAGGGAGTTAGAGTACAGCAG—2550-3′). The fragment was cloned into a pET30a (+) vector (Tiangen, Beijing, China) to construct the expression plasmids. The expression plasmid was transformed into *E. coli* BL_21_ competent cells (Tiangen); the truncated Hex protein was produced via isopropyl-β-D-thiogalactopyranoside (IPTG, GE Healthcare, Chicago, IL, USA) induction using pET30a- Hex *E. coli* at 37 °C. The truncated Hex protein was detected by SDS-PAGE and Western blotting (WB) analysis by anti-His mAb. Immunoreactive bands were verified by an enhanced chemiluminescence system (ECL; PerkinElmer Life Sciences, Fremont, CA, USA) [15]. The three-dimensional (3D) structure of the expressed protein was predicted with PyMOL software based on data from the SWISS-MODEL online server.

### 2.6. Biopanning of Phage Random Peptide Library against Anti-CAdV-2 mAbs

A commercially Phage Display Peptide Library Kit (New England BioLabs Inc., Ipswich, MA, USA) was used to carry out four rounds of biopanning according to the manufacturer’s manual. Briefly, 10 μg purified mAb 2C1 or 7D7 was coated in a 96-well plate and incubated overnight at 4 °C. The plates were washed with blocking buffer with 1% BSA at room temperature for 2 h and then washed 7 times with Tris-Buffered Saline and Tween 20 (TBST). Next, the phages in the phage library (M13) was diluted to 2 × 10^12^ PFU/mL and added to antibody-coated wells (100 μL/well), then incubated for 1 h at room temperature on the shaker. Bound phage were subsequently eluted with 100 μL of 0.2 M glycine/HCl (pH 2.2) containing 1 mg/mL BSA and the eluate was neutralized with 15 μL of 1 M Tris-HCl (pH 9.1) [19]. Eluted phage were amplified in *E. coli* (ER2738), and titrated on LB plates containing IPTG and X-Gal for the subsequent rounds of selection. The number of input phage was 10^11^ phages in subsequent each round of biopanning, too. After 4 rounds of screening [19,20], the eluted phages were plated and individual clones were picked randomly for phage ELISA as described [17,21].

### 2.7. DNA Sequencing and Displayed Peptide Analysis

The single phages with high absorbance were chosen for sequencing. DNA of phage clones was extracted using a DNA extraction kit (Tiangen, China), and sequenced with the 96 gIII sequencing primer (Shanghai Biological Engineering Technology Co. Ltd.; Beijing, China). The deduced aa sequences from the insert sequences were analyzed by the DNASTAR software and compared with Hex (AP_000622.1) from CAdV-2. In order to confirm the minimal epitopes recognized by 2C1 and 7D7 in Hex, a series of peptides spanning 4–10 aa of Hex with a purity greater than 95% (F1-F17) was synthesized (Shanghai Biological Engineering Technology Co. Ltd.; Beijing, China) as coating antigens for peptide ELISA assays (Table 1). 

### 2.8. Biological Information Analysis

To explore novel Hex epitope’s conservation among different CAdV strains and other adenovirus strains, aa sequences from all CAdV reference strains available in GenBank were compared with other adenovirus strains in the NCBI database (Table 2) using Megalign of DNASTAR software. The spatial distribution of the epitopes identified in Hex were analyzed by mapping epitope locations on a 3D model of CAdV-2 Hex (GenBank accession number AC000020.1) using PyMOL software based on results from the SWISS-MODEL online server. Hex secondary structural features were predicted using PROTEAN of DNASTAR software.

## 3. Results

### 3.1. Characterization of Two Neutralizing mAbs—2C1 and 7D7

Two mAbs, 2C1 and 7D7, against CAdV-2, were generated and classified by subtype to into the IgG1 subclass, and light chains were the κ-type (Table 3). Furthermore, a micro-neutralization assay on MDCK was used to determine the neutralizing activities of both mAbs. The mAb 2C1 could neutralize potently CAdV-2 with an NT50 of 4096, but mAb 7D7 could neutralize partly against CAdV-2 with an NT50 of 64 (Table 3).

**Figure 1:**
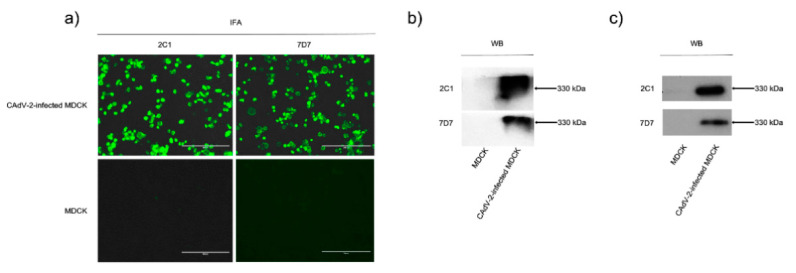
Identification of mAbs reacting with CAdV-2 by indirect immunofluorescence and Western blot. MDCK cells were infected with CAdV-2 at a multiplicity of infection (MOI) = 10 for 24 h, and mAbs 2C1 and 7D7 were used to detect the virus (**a**) by indirect immunofluorescence assay (magnification = 200×); (**b**) Western blot (WB) after native PAGE; and (**c**) WB after SDS-PAGE.

We confirmed that mAbs 2C1 and 7D7 reacted specifically with CAdV-2-infected MDCK cells in an indirect immunofluorescence assay (IFA) and native CAdV-2 protein in a WB after native PAGE (Figure 1a,b), and also bound purified CAdV-2 antigen in ELISA (Figure S1). Both mAbs could recognize denatured CAdV-2 proteins in WB (Figure 1c), indicating that they are binding to linear epitopes.

### 3.2. MAbs 2C1 and 7D7 Specifically Recognize the CAdV-2 Hexon Protein

**Figure 2:**
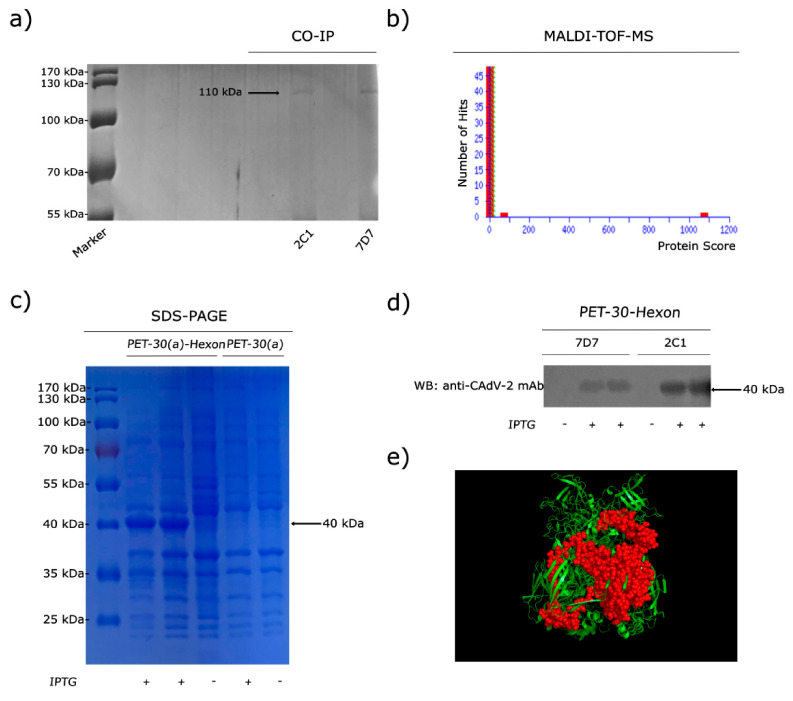
MAbs 2C1 and 7D7 react with the CAdV-2 hexon. (**a**) SDS-PAGE of CAdV-2 proteins after immunoprecipitation with mAbs 2C1 and 7D7. Marker: PageRuler Prestained Protein Ladder. (**b**) Results of MALDI-TOF-MS analysis. Protein scores (n = 1074 for mAbs) that were greater than 26 were significant (*p* < 0.05). Protein scores were derived from ion scores as a non-probabilistic basis for ranking protein hits. (**c**) A truncated, recombinant peptide spanning the C-terminal 300 aa of the CAdV hexon protein was expressed in *Escherichia coli*. Coomassie-stained SDS-PAGE showing characteristic 40-kDa band; marker: PageRuler Prestained Protein Ladder. (**d**) Reactivity of the truncated hexon protein with mAbs 2C1 and 7D7 by Western blot analysis. (**e**) 3D structure of the expressed 300 aa from the truncated peptide (red spheres) predicted by the SWISS-MODEL online server.

WB results showed that the mAbs 2C1 and 7D7 recognized a 330 kDa band after both native PAGE and SDS-PAGE of CAdV-2 (Figure 1b,c). In order to determine which protein was binding, virions were immunoprecipitated (IP) with mAbs 2C1 or 7D7. Immunoreactive protein bands were visualized in SDS-PAGE (Figure 2a), and immunoprecipitates were analyzed through MALDI-TOF-MS based on the peptide mass matching. By searching in the NCBI database, the reactive protein (protein scores 1074 larger than 26 were significant, *p* < 0.05) was identified as CAdV-1 Hex (taxon identifier: 69150), which has a molecular mass of 110 kDa (Figure 2b). Thus, the 330 kDa protein recognized in WB corresponds to the trimeric form of Hex, which was not reduced to monomeric Hex after 2-ME treatment, suggesting that the bonds between monomers are not disulfide bonds but non-covalent bonds. 

In order to define the domain of the protein binding to the mAbs, a truncated recombinant protein spanning the C-terminal 300 aa of the CAdV-2 Hex was generated, which is predominantly expressed in the form of inclusion body in *E. coli* after 0.5 mM IPTG induction. SDS-PAGE analysis revealed a 40-kDa band in the original expression products (Figure 2c). WB results showed that the truncated Hex peptide was specifically recognized by mAbs 2C1 and 7D7 (Figure 2d). A predicted 3D structure of truncated monomeric form of the Hex peptide was generated using PyMOL software on the basis of data predicted through the online server of SWISS-MODEL (Figure 2e).

### 3.3. Phage-Displayed Mimic Epitopes Biopanned by mAbs 2C1 and 7D7

**Figure 3:**
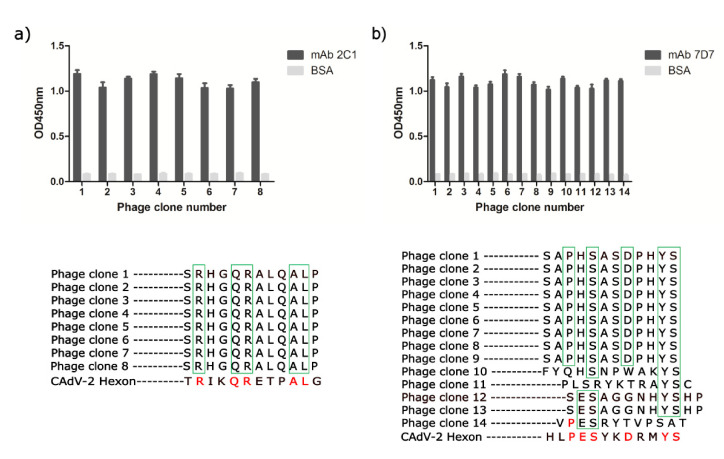
Confirmation of the epitope recognized by mAbs 2C1 and 7D7. Selected positive phage clones reacted specifically with mAb 2C1 and 7D7 in phage ELISA. Eight and fourteen phage clones were selected after four rounds of biopanning for (**a**) 2C1 or (**b**) 7D7, respectively, and their binding was analyzed by phage ELISA (upper). Three independent assays were carried out. Lower: sequence comparison of random peptide inserts displayed on the positive phages to the CAdV-2 sequence.

To determine the linear epitopes recognized by neutralizing mAbs 2C1 and 7D7, affinity purified mAbs 2C1 and 7D7 were used to perform phage-displayed 12-mer random peptide library biopanning. After four rounds of panning, an enrichment of phage that bound to mAbs 2C1 or 7D7 was achieved. 15 individual phage clones were isolated from the final eluted phage plate, and their reactivities to mAbs 2C1 or 7D7 were assessed by phage ELISA, with monophages exhibiting high absorbance selected for sequencing. Thus, 8 and 14 individual positive phage clones from 2C1 or 7D7, respectively, were selected for sequencing of inserts (Figure 3). All eight positive phage clones with high affinity to mAb 2C1 displayed an identical 12-mer peptide “SRHGQRALQALP,” similar to the sequence ^634^RIKQRETPAL^643^ observed in the CAdV-2 Hex (Figure 3a). The high-affinity phage clones with mAb 7D7 displayed a consensus motif PESXXDXXYS (X is any aa), similar to the sequence ^736^PESYKDRMYS^745^ observed in the CAdV-2 Hex (Figure 3b). 

**Figure 4:**
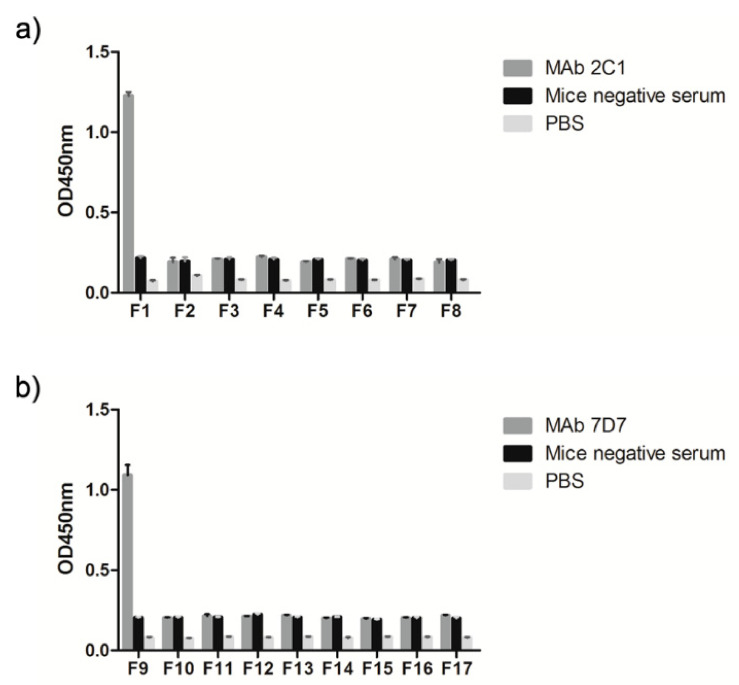
Truncated peptides define the minimal linear epitope recognized by mAb 2C1 or 7D7. (**a**) ELISAs were performed using synthetic peptides (4–10 aa) with different deleted aa residues from the N and/or C termini of the peptide ^634^RIKQRETPAL^643^ to determine the minimal linear epitope sequence recognized by mAb 2C1. (**b**) ELISAs were performed using synthetic peptides (4–10 aa) with deleted different aa residues from the N and/or C termini of the peptide ^736^PESYKDRMYS^745^ to determine the minimal linear epitope sequence recognized by mAb 7D7.

The preliminary localization of the linear neutralizing epitope recognized by 2C1 and 7D7 was verified using a panel of synthetic peptides of variable length (4–10 mers) to identify the minimal reactivity unit of each epitope. These 17 synthetic peptides as coating antigens were used to perform ELISAs, with an irrelevant protein and the recombinant 300-aa Hex fragment used as negative and positive controls, respectively. The result showed that peptides F1 and F9 were recognized by mAbs 2C1 and 7D7, respectively (Figure 4), suggesting that the core sequences ^634^RIKQRETPAL^643^ and ^736^PESYKDRMYS^745^ are the minimal required sequences for antibody binding.

### 3.4. Spatial Location of the Epitopes

**Figure 5:**
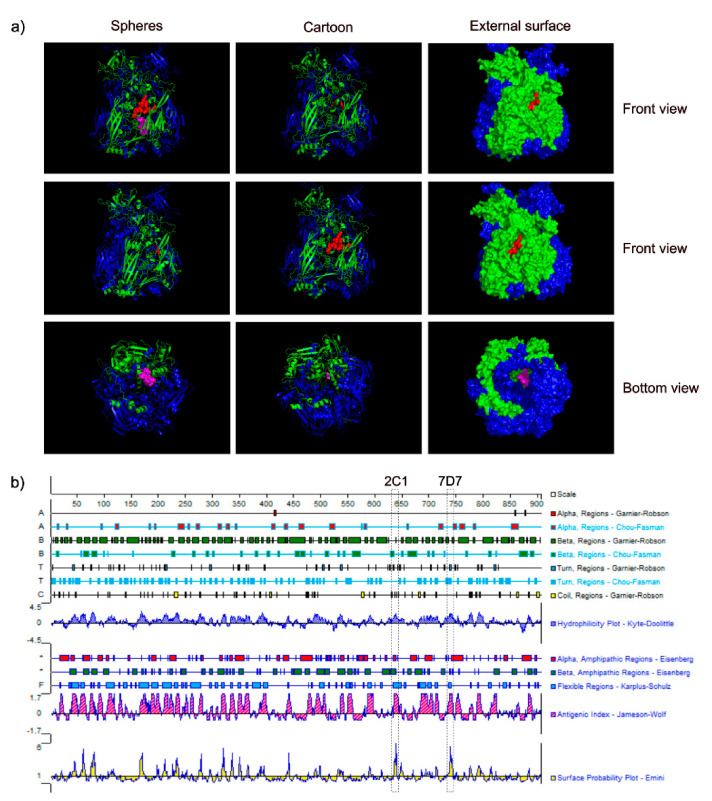
Spatial structure and positions of the identified epitopes. (**a**) Relative locations of the identified epitopes are presented in spherical, cartoon and external surface depictions of monomer hexon from a 3D structure of CAdV-2 trimeric hexon that was predicted using the SWISS-MODEL online service. Epitopes recognized by mAbs 2C1 and 7D7 are shown in red and purple, respectively, separately, and from the front view and the bottom view. (**b**) Secondary structure features of the CAdV-2 hexon were predicted using PROTEAN software. The putative 2C1 and 7D7 epitopes are shown in the boxes.

To understand the structure of two epitopes identified by mAbs 2C1 and 7D7, we studied the arrangement and localization of epitopes in the 3D structure of trimeric forms of Hex. This analysis revealed that the epitope of 2C1-recognized was exposed fully on the surface of the predicted CAdV-2 trimeric Hex structure, forming part of a beta-sheet (Figure 5a). In contrast, the epitope recognized by mAb 7D7 is located within the inner region of the predicted trimeric structure as part of a beta-sheet (Figure 5a), and is thus a cryptic epitope. Analysis using PROTEAN predicted that both epitope sequences should form part of a beta-sheet (Figure 5b), which is the same as the predicted 3D structure shown in Figure 5a. Furthermore, surface-exposed ^634^RIKQRETPAL^643^ is predicted to have strong hydrophilicity (the score: 2.04) and a high antigenic index (the score: 3), which suggested that it may be an important B-cell epitope on the CAdV-2 Hex.

### 3.5. Conservation of Novel Epitopes in Different AdV Strains

**Figure 6:**
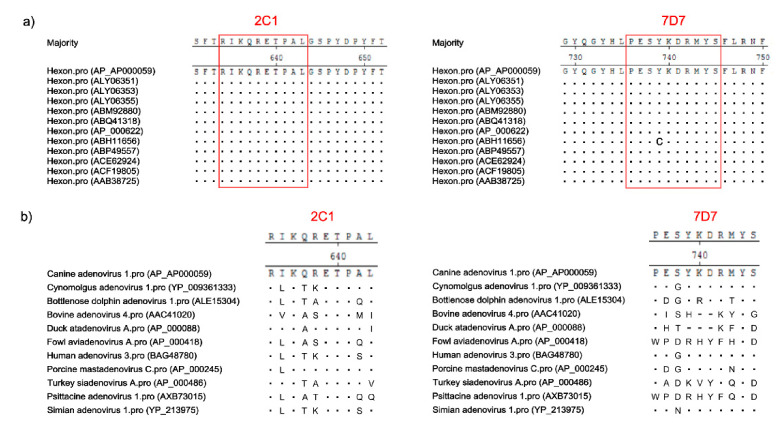
Amino acid sequence alignments of the epitope regions from several different AdV hexon proteins. (**a**) Sequence comparison of the mAb epitopes of different CAdV isolates available in GenBank. The homologous sequences corresponding to the putative hexon epitopes for mAbs 2C1 and 7D7 are shown in red boxes. (**b**) Alignment of the CAdV mAb epitope sequences with 10 other AdV hexon proteins available in GenBank.

To determine whether the two novel epitopes recognized by 2C1 or 7D7 are conserved among other AdV strains, the aa sequences of both epitopes were aligned with putative aa sequences from different AdV isolates available in GenBank. As shown in Figure 6a, the 2C1 linear epitope ^634^RIKQRETPAL^643^ was found in all CAdV Hex genes available in GenBank. The 7D7 epitope ^736^PESYKDRMYS^745^ was similarly conserved among the hexon proteins of all CAdV isolates available in GenBank, though one CAdV-2 strain (ABH11656) had a single aa polymorphism (Y- > C) (Figure 6a). However, neither epitope sequence was highly conserved in other different AdV isolates available in GenBank, except porcine mastadenovirus C had a single aa polymorphism (I- > L) in epitope ^634^RIKQRETPAL^643^; and cynomolgus adenovirus, human adenovirus 3 and simian adenovirus 1 had a single aa polymorphism (S- > G/N) in epitope ^736^PESYKDRMYS^745^ (Figure 6b).

## 4. Discussion

Viruses display various epitopes on their outer surface that generally play roles in antibody-mediated immune responses leading to protection. Mapping these epitopes as a function of their recognition by mAbs is a powerful tool for the diagnosis of diseases [22]. Effective protection against viral infection usually requires inducing a high level of neutralizing antibodies [23], which can recognize specific antigenic epitopes or sites located on the surfaces of viral particles. Thus, in the development of epitope-based vaccines, the first step is the identification of antigenic epitopes recognized by neutralizing mAbs. Neutralizing antigenic sites have been identified on many viruses, including foot-and-mouth disease virus, HAdV-14 and waterfowl parvoviruses [17,18,24,25]. However, to date there is a lack of information finely mapping the neutralizing epitopes on CAdV.

In the present study, the strongly CAdV-2 neutralizing mAb 2C1 and a partially neutralizing mAb 7D7 have been characterized in vitro. The passive immunization and protection test will be further investigated in future studies to determine the actual neutralizing effect in vivo. The results of biopanning a phage-display library provided clues for us to locate the true epitopes in monomeric Hex, and synthesized peptides further revealed the precise core determinants of the mAbs 2C1 and 7D7 binding sites were ^634^RIKQRETPAL^643^ and ^736^PESYKDRMYS^745^. The two epitopes are highly conserved among CAdV isolates, but not in other adenovirus isolates available in GenBank, which all have 1–9 aa differences (most have more than three aa differences). This suggests that two mAbs agaist two epitopes could be used simultaneously to develop a diagnostic method for CAdV in dogs.

The structural model showed that the identified B-cell epitope recognized by 2C1 existed on the surface of the trimeric Hex and formed part of a beta-sheet, which is in line with PROTEAN prediction. This information suggests that the 2C1-recognized epitope is likely an important epitope, which may allow us to develop CAdV epitopes vaccines and perhaps better induce neutralizing antibodies in immunized animals. Moreover, mAb 2C1 also can be used to develop therapeutic vaccines or antibody therapeutics as a result of its good neutralization ability regarding the virus. However, the 7D7-recognized B-cell epitope is present in the inner region of the trimeric Hex, although PROTEAN software predicted that this epitope was located on the surface of monomeric Hex. Thence, mAb 7D7 cannot be used to develop therapeutic vaccines or antibody therapeutics because of its weaker neutralization ability regarding the virus.

In our study, native trimers of CAV-2 Hex from whole virions and denatured trimers of CAV-2 Hex from virus lysates could both be detected by the mAbs, which suggests the mAbs recognized two linear epitopes. The trimeric Hex was shown to be resistant to the formation of monomeric chain units by 2-ME treatment under SDS-PAGE with boiling, which is consistent with previous reports [26]. It suggests that the connections between the monomer chains are through non-covalent bonds but not disulfide bonds. 

## 5. Conclusions

Two novel linear neutralizing epitopes on the Hex of CAdV were identified, which provides valuable data for the design of epitope-based vaccines or therapeutic vaccines, antibody therapeutics and diagnostic markers.

## Figures and Tables

**Table 1:** Sequences of peptides synthesized in this study.

Name	Peptide
F1 (634–643 aa)	RIKQRETPAL
F2 (635–642 aa)	IKQRETPA
F3 (636–641 aa)	KQRETP
F4 (636–640 aa)	KQRET
F5 (636–639 aa)	KQRE
F6 (637–642 aa)	QRETPA
F7 (638–642 aa)	RETPA
F8 (639–642 aa)	ETPA
F9 (736–745 aa)	PESYKDRMYS
F10 (737–744 aa)	ESYKDRMY
F11 (738–744 aa)	SYKDRMY
F12 (739–744 aa)	YKDRMY
F13 (739–743 aa)	YKDRM
F14 (739–742 aa)	YKDR
F15 (740–745 aa)	KDRMYS
F16 (741–745 aa)	DRMYS
F17 (742–745 aa)	RMYS

**Table 2:** Hexon sequences cited in this study.

Accession Number	Total Length (aa)	Adenovirus Type
AP000059.1	905	Canine adenovirus 1
KP840545.1	905	Canine adenovirus 1
KP840547.1	905	Canine adenovirus 1
KP840549.1	905	Canine adenovirus 1
EF206692.1	905	Canine adenovirus 1
EF559262.1	905	Canine adenovirus 1
AP000622.1	905	Canine adenovirus 2
DQ839392.1	905	Canine adenovirus 2
EF508034.1	905	Canine adenovirus 2
EU717145.1	905	Canine adenovirus 2
EU794687.1	905	Canine adenovirus 2
U77082.1	905	Canine adenovirus 2
YP_009361333	925	Cynomolgus adenovirus 1
ALE15304	942	Bottlenose dolphin adenovirus 1
AAC41020.2	910	Bovine adenovirus 4
AP_000088.1	910	Duck atadenovirus A
AP_000418.1	942	Fowl aviadenovirus A
BAG48780	944	Human adenovirus 3
AP_000245.1	910	Porcine mastadenovirus C
AP_000486.1	906	Turkey siadenovirus A
AXB73015.1	942	Psittacine adenovirus 1
YP_213975.1	931	Simian adenovirus 1

**Table 3:** Biological characterization of mAbs against CAdV-2.

mAb	Subclass	IFA	Neutralization Titer	Western Blot
2C1	IgG1/κ	++++	2731 ± 682.7 (n = 3)	+
7D7	IgG1/κ	++++	53.33 ± 10.67 (n = 3)	+

All mAbs were diluted from an initial dilution of 1:2; a sample was considered positive if the NT titer was ≥ 45; ++++, very strong reactivity in IFA; +, reactivity in Western blot.

## Data Availability

No new data were created or analyzed in this study. Data sharing is not applicable to this article.

## Supplementary Materials

The following are available online at https://www.mdpi.com/2076-393X/9/2/135/s1, Figure S1: mAbs 2C1 and 7D7 reacted specifically with purified CAdV-2 antigen in ELISA. The purified CAdV-2 was coated with 100 ng/well in the ELISA plate, mAbs 2C1; 7D7 ascetic fluid (1:10,000) was the first antibody; HRP-labeled goat anti-mouse IgG (1:10,000) was the second antibody; PBS was the negative control and mouse anti- CAdV-2 serum was the positive control for ELISA.
