# Supplementary material for: Identification of Two Novel Linear Neutralizing Epitopes within the Hexon Protein of Canine Adenovirus Using Monoclonal Antibodies
Source: Vaccines (Basel). 2021 Feb 8;9(2):135. doi: 10.3390/vaccines9020135 (PMC7914820; doi:10.3390/vaccines9020135)
Supplement: Supplementary file 1 [file vaccines-09-00135-s001.pdf]

vaccines

MDPI

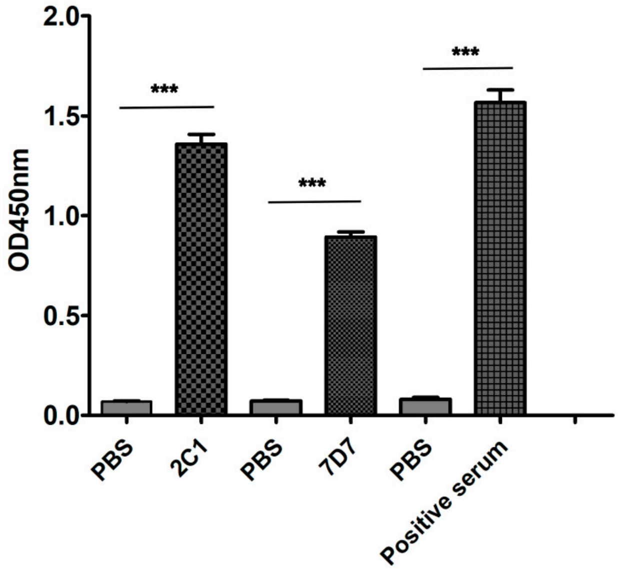

Figure S1. mAbs 2C1 and 7D7 reacted specifically with purified CAdV-2 antigen in ELISA. The purified CAdV-2 was coated with 100 ng/well in the ELISA plate, mAbs 2C1; 7D7 ascetic fluid (1:10000) was the first antibody; HRP-labeled goat anti-mouse IgG (1:10000) was the second antibody; PBS was the negative control and mouse anti- CAdV-2 serum was the positive control for ELISA.
